# Neurological Sequelae in Children Born After Assisted Conception Techniques in a Tertiary University Hospital in Riyadh, Saudi Arabia: A Retrospective Cohort Study

**DOI:** 10.7759/cureus.76908

**Published:** 2025-01-04

**Authors:** Fadi Busaleh, Fahad A Bashiri, Ahmed A Almasabi, Aljoharah A Almaziad, Alhanouf Alhaluli, Taif Alshammari, Abdullah E Bu Saleh, Abdulhadi A Alali

**Affiliations:** 1 Pediatrics, Maternity and Children Hospital (MCH), Al-Ahsa, SAU; 2 Pediatrics/Pediatric Neurology, King Saud University College of Medicine, Riyadh, SAU; 3 Pediatric Neurology, King Saud University Medical City (KSUMC), Riyadh, SAU; 4 Medicine, Majmaah University, Riyadh, SAU; 5 Medicine, King Saud University Medical City (KSUMC), Riyadh, SAU; 6 Pediatrics, King Fahad Medical City, Riyadh, SAU; 7 Orthopedics and Rehabilitation, Al Jafar General Hospital, Al-Ahsa, SAU; 8 Emergency, Al Jafar General Hospital, Al-Ahsa, SAU

**Keywords:** assisted conception, intracytoplasmic sperm injection (icsi), in vitro fertilization (ivf), low birth weight, neonatal intensive care unit (nicu), neurological outcomes, pediatric health, preterm birth, reproductive technology, systemic complications

## Abstract

Background

Assisted conception (AC) methods, including in vitro fertilization (IVF) and intracytoplasmic sperm injection (ICSI), have transformed reproductive medicine by offering solutions to infertility. However, their long-term health implications, particularly on neurological outcomes in children, require further investigations, especially in the Middle East.

Objective

This study aimed to evaluate the neurological complications among children conceived through assisted reproductive techniques at King Khalid University Hospital, a tertiary hospital in Riyadh, Saudi Arabia.

Methods

A descriptive retrospective review was conducted on the records of pediatric patients born via assisted conception between January 2017 and December 2022. Eligible children aged 18 months and older were included. Data on demographic characteristics, antenatal and postnatal complications, and neurological assessments were collected and analyzed using the Statistical Package for Social Sciences (SPSS) version 21 (IBM Corp., Armonk, NY).

Results

Out of 303 patients, 283 children met the inclusion criteria, with a mean age of 4.1 ± 1.7 years. Most deliveries were via cesarean section (CS) (194, 68.6%). Premature births accounted for 129 (45.6%), with antenatal complications reported in 58 (45.0%). The primary neurological sequela identified was speech disorder, affecting 18 children (6.4%), with a smaller proportion experiencing behavioral issues (eight, 2.8%) and motor delays (five, 1.8%). Significant associations were found between neurological complications and factors such as very preterm status, low birth weight (LBW), neonatal intensive care unit (NICU) admission, and associated complications during NICU stays.

Conclusion

Although the majority of children conceived through assisted reproductive techniques exhibit no significant complications, those born prematurely appear to have a higher risk of neurological issues. These findings underscore the importance of ongoing monitoring for children born through assisted conception, particularly those with identified risk factors. Future prospective studies are necessary to further elucidate these associations and improve clinical outcomes for this population.

## Introduction

Assisted conception (AC) was developed in the late 20th century and is provided through two primary methods: in vitro fertilization (IVF) and intracytoplasmic sperm injection (ICSI). These emerging and increasingly utilized methods of conception have been associated with certain maternal and fetal morbidities and mortalities. Examples of early complications include an increased incidence of multiple pregnancies (twins, triplets, or quadruplets), premature deliveries, and the need for assisted delivery, compounded by maternal risks associated with advanced age [1,2].

Early research predominantly focused on immediate complications observed in neonates or mothers. However, the long-term adverse health consequences, particularly neurological ones, have been less frequently studied [3].

Adverse long-term neurological sequelae in children are diverse, ranging from organic defects, such as congenital structural anomalies of the central or peripheral nervous system, to cellular-level disorders, including metabolic conditions. These findings have prompted researchers to investigate potential differences between children conceived through AC and those conceived naturally [4,5].

Although AC techniques have continued to advance, local literature from Saudi Arabia and the Middle East remains limited concerning the neurological sequelae observed following such procedures. This study aims to address this gap by examining the potential links between AC and adverse neurological outcomes.

## Materials and methods

Study design

A descriptive retrospective record review was conducted using King Khalid University Hospital's electronic file system. Data were collected by reviewing reports of pediatric patients born after AC between January 2017 and December 2022. Patients' files were accessed through the databases of the obstetric delivery book by identifying pregnant women who conceived through AC methods at King Khalid University Hospital, a tertiary care center in Riyadh, Saudi Arabia. Ethical approval for the study was obtained from the Institutional Review Board at King Saud University with reference number 24/1108/IRB.

Study population

A total of 303 patients who were delivered after assisted conception methods were enrolled in the study.

Inclusion criteria

To ensure an accurate neurological diagnosis, only children aged 18 months or older at the time of the study, who were delivered at King Khaled University Hospital, King Saud University, Riyadh, between January 2017 and December 2022, were included.

Exclusion criteria

Patients younger than 18 months at the time of the study or those with neurological or behavioral diseases caused by trauma or intoxication were excluded. Additionally, patients with incomplete files or those who died before reaching 18 months of age were excluded.

Two groups of outcomes were assessed. The first focused on the neonatal period (primary outcomes), and the second focused on outcomes after the neonatal period (secondary outcomes). These were evaluated based on the 10th edition of the International Classification of Diseases for neurological and behavioral diseases.

For the primary outcomes, the variables studied included demographic data (e.g., age and gender), antenatal complications for both fetus and mother, the mode of delivery, birth weight, gestational age (Table 1), the need for neonatal intensive care unit (NICU) admission, early neonatal complications, and any central nervous system structural abnormalities [6].

**Table 1:** Range definitions for birth weight and gestational age according to the World Health Organization.

Gestational age definition	
Term	≥37 weeks
Preterm	<37 weeks
Very preterm	<32 weeks
Extremely preterm	24-28 weeks
Birth weight criteria	
Normal birth weight	≥2500 g
Low birth weight	<2500 g
Very low birth weight	<1500 g
Extremely low birth weight	<1000 g

The secondary outcome variables included the presence or absence of epilepsy, motor or speech delay, behavioral disorders (e.g., attention deficit hyperactivity disorder {ADHD} and autism spectrum disorder {ASD}), and other neurological and general systemic complications.

Data analysis

The data were collected, reviewed, and analyzed using the Statistical Package for Social Sciences (SPSS) version 21 (IBM Corp., Armonk, NY). All statistical methods were two-tailed, with an alpha level of 0.05, and significance was considered if the p-value was less than or equal to 0.05.

Descriptive analysis was performed to summarize frequency distribution and percentages for children's biodemographic data and antenatal and postnatal assessments. Adverse neurological outcomes and other systemic outcomes of the children were also tabulated. The overall prevalence of adverse neurological sequelae among the study children was presented graphically.

Cross-tabulation was conducted to assess factors associated with neurological complications in children born after assisted conception using the Pearson chi-square test and the exact probability test for small frequency distributions.

## Results

Out of a total of 303 assisted conception techniques delivered, 20 cases were excluded, including five children who died before reaching 18 months of age and 15 due to incomplete files. This left 283 cases for analysis, consisting of 143 (50.5%) girls and 140 (49.5%) boys. Children's ages ranged from 18 months to seven years, with a mean age of 4.1 ± 1.7 years old. As for the mode of delivery, it was emergency cesarean section (CS) among 109 children (38.5%), elective CS among 85 (30%), spontaneous vaginal delivery among 83 (29.3%), and assisted delivery among six (2.1%). A total of 154 (54.4%) neonates were born full term, 83 (29.3%) were preterm infants, 29 (10.2%) were very preterm infants, and 17 (6%) were extremely preterm infants. Regarding antenatal complications, the most reported included gestational diabetes mellitus (54, 19.1%), hypothyroidism (30, 10.6%), intrauterine growth restriction (18, 6.4%), the premature rupture of the membrane (14, 4.9%), and per-vaginal bleeding (11, 3.9%). Most neonates (155, 54.8%) had no antenatal complications (Table 2).

**Table 2:** Bio-demographic characteristics and antenatal data of children born after assisted conception.

Bio-demographic and antenatal/postnatal data	Number	Percentage
Age in years		
<2 years	68	24.0%
2-5 years	136	48.1%
6-7 years	79	27.9%
Gender		
Male	140	49.5%
Female	143	50.5%
Mood of delivery		
Assisted delivery	6	2.1%
Elective cesarian section	85	30.0%
Emergency cesarian section	109	38.5%
Spontaneous vaginal delivery	83	29.3%
Gestational age classification		
Extremely preterm infant	17	6.0%
Very preterm infant	29	10.2%
Preterm infant	83	29.3%
Full-term infant	154	54.4%
Antenatal complication		
None	155	54.8%
Gestational diabetes mellitus	54	19.1%
Hypothyroidism	30	10.6%
Others	28	9.9%
Intrauterine growth restriction	18	6.4%
Premature rupture of the membrane	14	4.9%
Per-vaginal bleeding	11	3.9%

As for birth weight, it was normal among 141 (49.8%) neonates, low birth weight (LBW) among 113 (39.9%), very low birth weight (VLBW) among 19 (6.7%), and extremely low birth weight (ELBW) among 10 (3.5%). Exactly 102 (36%) neonates needed neonatal intensive care (NICU) admission, 29 (10.3%) had respiratory distress syndrome in NICU, six (2.1%) had central nervous system anomalies, three (1.1%) had sub-galeal hemorrhage, three (1.1%) had intraventricular hemorrhage, and three (1.1%) had retinopathy of prematurity (ROP). Most of the neonates (84.4%) had no neonatal complications. The mean Apgar score at five minutes was 7.5 ± 1.1, which increased to 8.8 ± 0.6 at 10 minutes (Table 3).

**Table 3:** Neonatal assessment of children born after assisted conception, including early complications. NICU: neonatal intensive care unit

Neonatal assessment	Number	Percentage
Birth weight		
Normal	141	49.8%
Low birth weight	113	39.9%
Very low birth weight	19	6.7%
Extremely low birth weight	10	3.5%
NICU admission		
No	181	64.0%
Yes	102	36.0%
NICU complications and neurological outcomes		
None	247	87.9%
Respiratory distress syndrome	29	10.3%
Central nervous system anomaly	6	2.1%
Others	5	1.8%
Neonatal Jaundice	4	1.4%
Sub-galeal hemorrhage	3	1.1%
Retinopathy of prematurity	3	1.1%
Acquired central nervous system insult	2	0.7%
Necrotizing enterocolitis	2	0.7%
Sepsis	2	0.7%

A total of 18 (6.4%) children had speech disorders, eight (2.8%) had behavioral disorders, five (1.8%) had motor delay, two (0.7%) had seizures beyond the neonatal period, two (0.7%) had hearing disorders, and one (0.4%) had other neurological diseases (Table 4).

**Table 4:** The postnatal neurological sequelae among children born after assisted conception.

Neurodevelopmental complications	Yes		No	
	Number	Percentage	Number	Percentage
Speech disorder	18	6.4%	265	93.6%
Behavioral disorder	8	2.8%	275	97.2%
Central nervous system anomaly	6	2.1%	277	97.9%
Motor delay	5	1.8%	278	98.2%
Seizure disorder	2	0.7%	281	99.3%
Hearing disorder	2	0.7%	281	99.3%
Other neurological diseases	1	0.4%	282	99.6%

Only two (0.7%) neonates had a chromosomal anomaly, 21 (7.4%) had respiratory complications, 17 (6%) had dermatological diseases, 16 (5.7%) had cardiological complications, 12 (4.2%) had gastrointestinal complications, 10 (3.5%) had genitourinary complications, and nine (3.2%) had musculoskeletal complications (Table 5).

**Table 5:** Systematic complications among children born after assisted conception rather than the neurological one.

System involved	Number	Percentage
Respiratory	21	7.4%
Dermatology	17	6.0%
Cardiology	16	5.7%
Gastrointestinal	12	4.2%
Genitourinary	10	3.5%
Musculoskeletal	9	3.2%
Ear, nose, and throat	7	2.5%
Renal	5	1.8%
Endocrinology	5	1.8%
Hematology	4	1.4%
Ophthalmology	3	1.1%

**Figure 1:**
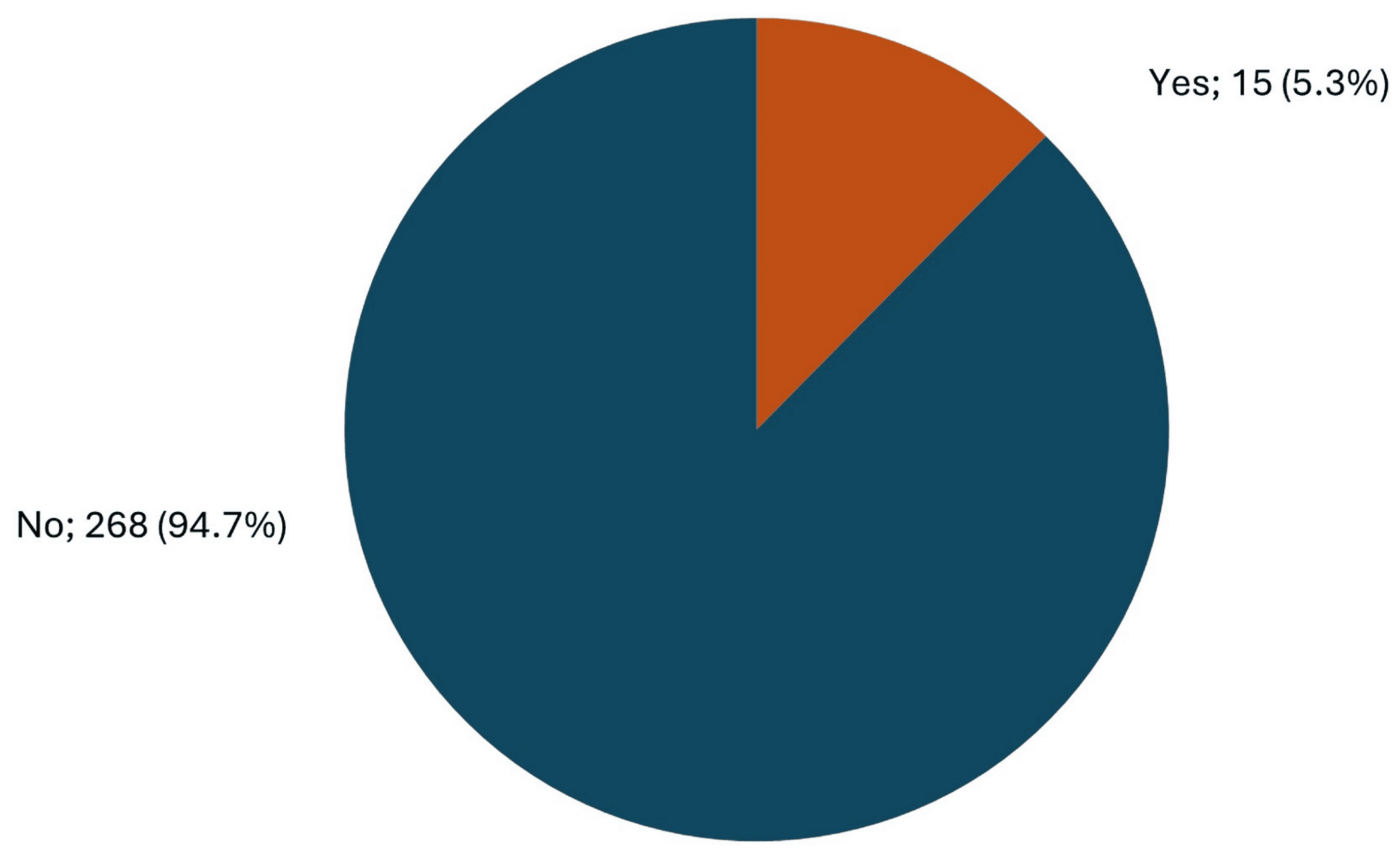
The overall prevalence of neurological sequelae among children born after assisted conception.

A total of 15 (5.3%) neonates had any of the neurological complications, while the vast majority (268, 94.7%) had none of these complications (Figure 1).

Exactly 24.1% of very preterm infants had neurological complications versus 11.7% of full-term infants with a recorded statistical significance (P = 0.049). Also, 31.6% of VLBW infants had neurological complications in comparison to 11.3% of others with normal birth weight (P = 0.013). Neurological complications were detected among 17.6% of neonates who needed NICU admission compared to 9.4% of those who did not (P = 0.043). Likewise, 20.6% of neonates with NICU complications had neurological complications compared to 11.2% of others without (P = 0.049) (Table 6).

**Table 6:** Factors associated with neurological complications among children born after assisted conception. *Significant p-value, less than 0.05. NICU: neonatal intensive care unit

Factors	Adverse neurological sequalae				P-value
	Yes		No		
	Number	Percentage	Number	Percentage	
Age in years					0.516
<2 years	11	16.2%	57	83.8%	
2-5 years	16	11.8%	120	88.2%	
6-7 years	8	10.1%	71	89.9%	
Gender					0.091
Male	22	15.7%	118	84.3%	
Female	13	9.1%	130	90.9%	
Mood of delivery					0.508
Assisted delivery	0	0.0%	6	100.0%	
Elective cesarian section	9	10.6%	76	89.4%	
Emergency cesarian section	17	15.6%	92	84.4%	
Spontaneous vaginal delivery	9	10.8%	74	89.2%	
Gestational age classification					0.049*
Extremely preterm infant	4	23.5%	13	76.5%	
Very preterm infant	7	24.1%	22	75.9%	
Preterm infant	6	7.2%	77	92.8%	
Full-term infant	18	11.7%	136	88.3%	
Antenatal complication					0.250
Yes	19	14.8%	109	85.2%	
No	16	10.3%	139	89.7%	
Birth weight					0.013*
Extreme low birth weight	3	30.0%	7	70.0%	
Very low birth weight	6	31.6%	13	68.4%	
Low birth weight	10	8.8%	103	91.2%	
Normal	16	11.3%	125	88.7%	
NICU admission					0.043*
No	17	9.4%	164	90.6%	
Yes	18	17.6%	84	82.4%	
NICU complications					0.049*
Yes	7	20.6%	27	79.4%	
No	28	11.2%	221	88.8%	

## Discussion

Assisted conception techniques, developed in the late 20th century, have revolutionized the field of reproductive medicine, providing hope to couples struggling with infertility. The two primary methods of assisted conception are in vitro fertilization (IVF) and intracytoplasmic sperm injection (ICSI). These advanced reproductive technologies have enabled countless individuals and couples to achieve pregnancy when natural conception has proven difficult or impossible [7,8]. However, the long-term health consequences, particularly neurological outcomes, have been less frequently studied, especially in the Middle East.

Our study has investigated different aspects of possible complications that can be observed by focusing on the neurological one.

Primary outcomes

The primary neonatal outcomes highlighted several key findings. The distribution of delivery modes showed a higher prevalence of assisted delivery techniques, with cesarean section being the most common delivery method, accounting for more than two-thirds of deliveries. This reflects the need for advanced medical interventions in pregnancies involving assisted conception.

In addition, around half of the delivered babies were preterm, which may serve as an additional risk factor for both assisted delivery techniques and the need for NICU admission and its associated complications. Antenatal complications were present in nearly half of the neonates and were primarily related to maternal health conditions rather than fetal issues. These factors make these neonates more vulnerable to a variety of complications.

Despite the high prevalence of antenatal and neonatal complications, most neonates did not experience significant neonatal issues, suggesting the effective management of these high-risk pregnancies and advancements in neonatal care. Notably, no significant neurological disorders were observed during the neonatal period, and most issues were sequelae of preterm complications. This aligns with findings from various studies, as the risk of complications in neonates conceived through assisted conception was not markedly different from those conceived naturally. However, some literature reports a higher prevalence of neurological sequelae, which may be attributed to the technical aspects of assisted conception or specific genetic diagnoses [9-11].

In comparison to a local study conducted in Saudi Arabia by Alghadier et al., it was noted that in the general population, respiratory complications are the most significant issues observed in preterm infants, with neurological complications ranked among the top five [10]. This is somewhat similar to our findings, as respiratory complications were highly prevalent and represented the most affected system. However, neurological adverse events were less frequent in our findings.

In our research, we observed congenital central nervous system complications more frequently than acquired preterm complications, such as intraventricular hemorrhage. Finally, our findings are in agreement with Alghadier et al., highlighting that extreme prematurity and low birth weight are the leading factors contributing to subsequent complications [10].

Secondary outcomes

The secondary outcomes focused on longer-term neurological and systemic complications. Neurological sequelae were observed in a small percentage of children, with speech disorders being the most common. Behavioral disorders, motor delays, and seizures were less prevalent, in descending order. These findings align with some existing literature suggesting that, in general, there is no significant difference in neurological complications between naturally conceived children and those conceived through assisted reproduction. Interestingly, some studies even suggest a lower incidence of complications in the latter group.

What stands out in our findings is the prominence of speech disorders as the primary neurological complication, differing from other studies that emphasize behavioral issues as the most frequently encountered problem. For instance, behavioral complications, including autism spectrum disorder (ASD), have often been highlighted in other research as in Ahmadi et al. [11], and even though the median age for ASD diagnosis, as noted in local studies by Alnemary et al. [12] and AlBatti et al. [13], is typically between two and five years, which was also the largest age group in our study, ASD was not the most commonly observed complication in our research.

On the other hand, the prevalence of speech problems in our findings could be seen as an early marker for autism. The delay in diagnosing ASD in our cohort may be related to several factors. As suggested by Alenezi et al., there is a significant gap in Saudi Arabia between the need for individualized diagnostic services for autism and the availability of such services, which contributes to delays in diagnosis [14]. Another contributing factor is that our study included children up to early school age (seven years), potentially missing late-onset neurodevelopmental outcomes that might manifest in older age groups.

In terms of neurogenetic complications, these were virtually absent in our cohort. This may be attributed to the early genetic testing performed during the fertilization process in assisted reproduction, which likely excluded any embryos with genetic susceptibilities [1,9,12-18].

Systemic complications

Systemic complications were also evaluated, with respiratory and dermatological complications being the most frequent. Cardiological, gastrointestinal, genitourinary, and musculoskeletal complications were less common but still noteworthy. These findings are similar to those observed in global literature on naturally conceived children [17].

Factors associated with neurological complications

Several factors were significantly associated with an increased risk of neurological complications.

Gestational Age

Very preterm infants exhibited a higher prevalence of neurological complications (24.1%) compared to full-term infants (11.7%). This highlights gestational age as a critical determinant of neonatal outcomes.

Birth Weight

Infants with very low birth weight (VLBW) had a higher incidence of neurological complications (31.6%) compared to those with normal birth weight (11.3%).

NICU Admission

Neonates requiring NICU care had a higher prevalence of neurological complications (17.6%) compared to those who did not require NICU admission (9.4%). Moreover, neonates who experienced complications during their NICU stay were more likely to develop neurological issues (20.6%) compared to those without complications (11.2%).

The analysis of these factors emphasizes the significant role of both antenatal and postnatal care in predicting neurological sequelae. These findings are consistent with previous local research, such as that by Alghadier et al. [10], as well as global studies [18-21].

Limitations

Several limitations must be acknowledged in this study. The retrospective design inherently carries the risk of missing or incomplete data, which could influence the findings. Additionally, the study was conducted at a single tertiary care center, which may limit the generalizability of the results to other settings or populations. The study population involved children of early school age, with limited inclusion of older children and insufficient focus on cognitive and school difficulties as part of neurological complications. Finally, the exclusion of children with incomplete records may have introduced selection bias.

## Conclusions

This study provides valuable insights into the health outcomes of children conceived through assisted reproductive methods. While the majority of these children do not experience significant complications, a subset remains at increased risk for neurological and systemic issues, particularly those who encounter significant morbidities early on in life after birth. These findings underscore the importance of close monitoring and follow-up for children conceived through assisted reproduction, especially those with identified risk factors. Further research, including prospective studies, is needed to confirm these findings and to develop targeted interventions to improve outcomes for this vulnerable population.
